# The Effect of Exercise Training on Quality and Quantity of Sleep and Lipid Profile in Renal Transplant Patients: A Randomized Clinical Trial

**Published:** 2014-11-01

**Authors:** S Pooranfar, E Shakoor, MJ Shafahi, M Salesi, MH Karimi, J Roozbeh, M Hasheminasab

**Affiliations:** 1 Physical Education Department, Shiraz University, Shiraz Iran; 2 Shiraz Transplant Research Center, Nemazee Hospital, Shiraz, Iran; 3 *Shiraz Nephrology/Urology Research Center, Shiraz University of Medical Sciences, Shiraz, Iran*

**Keywords:** Exercise training, Sleep quality, Sleep quantity, Lipid profile, Renal transplantation

## Abstract

Background: Patients undergoing renal transplantation consume immunosuppressive drugs to prevent graft rejection. Cardiovascular complications and reduced quality of sleep are among the side effects of these drugs. Studies have indicated that the use of non-therapeutic methods such as exercise is important to reduce these complications.

Objective: To evaluate the effect of a period of exercise training, as a non-therapeutic method, on quality and quantity of sleep and lipid profile in renal transplant patients.

Methods: 44 renal transplant recipients were selected to participate in the study and randomized into exercise (n=29) and control (n=15) groups. The exercise group participated in a cumulative exercise program 3 days a week for 10 weeks in 60–90-minute exercise sessions. Control group subjects did not participate in any regular exercise activity during this period. Sleep quality of the subjects was evaluated using Pittsburgh Sleep Quality Index (PSQI) questionnaire; the sleep quantity was assessed by recording the duration of convenient nocturnal sleep of the subjects. Physiological sleep-related variables (serum triglyceride [TG], and total, high-density lipoprotein [HDL], and low-density lipoprotein [LDL] cholesterol) were measured before and after 10 weeks of exercise training

Results: In exercise training group, sleep quality of the subjects was improved by 27%; the sleep quantity was increased by 30 minutes (p<0.05). TG, cholesterol and LDL values were significantly (p<0.05) decreased after 10 weeks of exercise training in the exercise group compared to the control group, however, no change was observed in serum HDL level in exercise group compared to the control. There was also a significant (p=0.05) difference in sleep quality and quantity between control and exercise groups. However, there was no correlation between changing quality and quantity of sleep with sleep-related physiological factors.

Conclusion: 10 weeks of exercise activity improved the quality and quantity of sleep as well as a number of sleep-related physiological parameters in renal transplant recipients, and would be an effective approach to treat sleep-related disorders in renal transplant recipients.

## INTRODUCTION

Chronic renal disease is a main general health problem worldwide [1]. Hemodialysis and renal transplantation are the main therapeutic methods for patients with end-stage renal disease [2]. Renal transplantation is now considered an effective approach in treatment of chronic renal failure [3]. Prevention of acute graft rejection is an important problem after renal transplantation. Renal transplant recipients require several immunosuppressive drugs, each with its own advantages and disadvantages [4]. Obesity, muscle waste, loss of hair, cardiovascular complications, insomnia and reduced quality of sleep are among common complications of these drugs. Sleep-related complaints have also been reported in more than 80% of patients with chronic renal failure [5].

Successful renal transplantation is expected to correct most abnormalities caused by chronic kidney disease (CKD) and significantly improve the patients’ health-related quality of life (HRQoL) [6, 7], but some studies have demonstrated that sleep disorders do not always improve after transplantation [8, 9].

The sleep quality and quality of life of kidney transplant recipients are known to be affected by several factors such as socio-demographic variables, comorbidities, psychiatric disorders, and other physical conditions, including tobacco use, uremia, malnutrition, and anemia [10]. Sleep-related disorders have become a focus of interest for research due to increasing evidence of an association between cardiovascular disturbances and sleep apnea-hypopnea syndrome [11, 12]. In addition, studies have suggested that sleep disorders are closely linked to physical, psychological and social well-being [13] and cognitive function [14, 15].

Sleep is a prerequisite with a high importance in human life. Without sufficient sleep, concentration capacity, sound judgment and daily activities are decreased, and irritability is increased [16]. Sleep disorder is a condition characterized by a disrupted sleep pattern or sleep-related behavior [17]. Age, physical activities, alcohol and caffeine containing drinks, diet and some specific diseases such as chronic renal failure can affect sleep quality and quantity [18]. Disturbed quality of sleep is associated with physical, behavioral and mental problems, and can lead to disrupted mental and social performance as well as interpersonal interactions [19].

On the other hand, any disturbance or reduction in duration of sleep may alter appetite hormones and increase the tendency of the individual for excessive consumption of high-calory foods, which seems to be indirectly related to serum lipid profile—serum high-density lipoprotein (HDL), triglyceride (TG), and total cholesterol (TC) [20]. In a cross-sectional study on 8860 persons, a significant correlation was observed between the lipid profile (TC, TG, HDL) and duration of sleep; those with higher TG and TC and lower HDL levels had a shorter sleep duration than those with normal levels of these lipids [20]. Investigations have shown that increased level of lipids and lipoproteins in serum is a risk factor of cardiac attack [21]. Scientific evidence has indicated short sleep duration as a risk factor for cardiac attack [22].

Use of drugs is the most common way to confront sleep problems. Non-pharmacological methods have a slower course of action than soporific drugs, however, they are long-lasting and lack the side effects of drugs such as addiction. Physical activity is a non-pharmacological method causing tranquility and increased body temperature, and is useful for sleep start and maintenance [23]. In the study of Alawski (2007), the sleep quality of the subjects was significantly improved after four weeks of hiking practice with moderate intensity [24]. Caldwell, *et al*, obtained good results for improvement in sleep quality in assessment of the effect of pilot exercises on sleep pattern of 18–32-year-old young men [25]. The results of another study showed that the general score of sleep quality questionnaire of Pittsburgh was significantly improved after 12 weeks of hiking [26]. The exercise program causes major changes in composition of body (fat content and lean body weight), which presumably entails reduced incidence of heart attack [21]. In view of life style modification, exercise and sports is a main approach to reduce cholesterol and promote health [27].

On the other hand, there is a significant correlation between sleep duration and physiological parameters including serum HDL, TC and TG [20, 28], so that there is a negative correlation between sleep duration and these parameters—sleep duration is decreased by increase in TC and TG levels. There was also a significant positive correlation between HDL and less than five hours of sleep, so that reduced sleep duration decreased the level of HDL in serum [20]. Altena, *et al*, showed that aerobic exercise five times a week for four weeks with 75% maximum heart rate causes decreased plasma TC and LDL levels and increased plasma HDL level [29]. Studies have indicated that moderate intensity physical activity for at least two months causes reduction in LDL level and increase in HDL level [30-33].

To the best of our knowledge, no study has so far been conducted to evaluate the correlation between exercise and sleep quality and quantity and lipid profile in renal transplant recipients. We therefore conducted this study to assess the effect of a period of exercise on sleep quality and quantity as well as lipid profile in renal transplant recipients.

## PATIENTS AND METHODS

In this clinical trial (before-after design) we had two arms—treatment group and control. Forty-four renal transplant recipients referred to Namazi Hospital, Shiraz, southern Iran, aged between 20 and 50 years were found eligible to participate in the study. Patients who received transplants 2–3 years before, had no history of consumption of alcohol and caffeine and had no regular exercise activities were included in this study. They were randomized into two arms of exercise (n=29) and control (n=15). After selection, the subjects participated in an orientation session. The exercise type and program and the likely results were described for participants; they were acquainted with cycling method on bicycle ergometer, treadmill and other exercises. The exercise program was designed by the researcher under supervision of a sports physiology expert and a specialist physician in terms of type, intensity, rehearsal and frequency after initial study and security, performance and facility checks according to physical status of the patients. The subjects in exercise group participated in the designed exercises for three 60–90-minute sessions per week for 10 weeks. The sessions were divided to three stages of pre-warming, main step, and rest. The pre-warming included stretching exercise by various body parts for 10–15 minutes. The main step began in a circle with a combination of aerobic and resistive exercises using ergometer bicycle, treadmill and free weights (aerobic exercise on fixed bicycle or treadmill with 40%–70% maximum heart rate intensity and resistive exercise with 45%–65% of maximum frequency). There were 9–17 stations, 3–6 circles in each session, relaxation time of 1–2 minutes between each station and 3–5-minute gaps between circles. At the end of each session, the “rest stage” was conducted by running with a slow pace, stretching movements and gentle exercises for 10 minutes. The control group experienced no regular physical exercise during the 10 weeks of the study. Research variables were measured at the end of exercise period in a similar way. Five mL of participants’ blood was drawn from the antecubital vein after 12 to 14 hours of fasting before and after 10 weeks of exercise. Sleep quality and quantity questionnaire was completed before and after the research.

Sleep quality protocol

Sleep quality was estimated using Pittsburg Sleep Quality Index (PSQI) questionnaire. It is a self-completed questionnaire evaluating the quality of sleep during the last month, and includes 19 questions in 7 parts (mental sleep quality, late sleep, sleep sufficiency, sleep period, sleep disorder, use of soporific drugs and defective function during the day). Each part can be scored from ‘0’ to ‘3.’ The total score of PSQI ranges between ‘0’ and ‘21,’ with higher scores indicating lower quality sleep. A score higher than ‘5’ indicates low quality sleep and many problems in at least two or more than three dimensions of this index [34]. In the study of Qoreyshi, *et al*, PSQI was found reliable (Cronbach’s α 0.83) [35].

Sleep quantity protocol

Sleep quantity of the participants was evaluated by their beneficial sleep period per minutes before and after the exercise program. Duration of beneficial sleep indicates the period of rest in the bed minus the total wide-awake period during the night. For this purpose, data about the sleep quantity of the subjects were recorded before and after the experience, and their beneficial sleep period was calculated accordingly.

The questions were as follows: “When they went to bed to sleep?” “At what time did they wake up?” “What was the period between their going to bed and their falling asleep?” “How many times during the night did they wake up?” “How much time was needed to go to bed again after they woke up?”

Statistical analysis

The data were analyzed by SPSS^®^ for Windows^®^. Descriptive statistics, *Student’s t* test were used for data analysis. To assess the correlation between sleep quality and quantity changes with changing values of TC, LDL, TG and HDL after 10 weeks of exercise, Pearson’s correlation coefficient was used.

## RESULTS

The mean±SD age, weight and medical history of participants were 36.2±2.2 years, 62.9±10.6, and 10.4±1.6 years, respectively. The mean overall PSQI score in exercise group was decreased from 7.21 to 5.26—27% improvement in sleep quality of the participants; their sleep quantity was increased by 30 minutes (p<0.05). However, in the control group, the mean overall PSQI score was increased from 9.12 to 9.89—7.7% reduction in the sleep quality; their sleep quantity was decreased by seven minutes, none of which was statistically significant.

There were significant differences between exercise and control groups in terms of sleep quality (p=0.037) and quantity (p=0.005), serum TG (p=0.001), TC (p=0.041) and LDL (p=0.007); there was no significant difference in HDL concentration (p=0.728) (Table 1).

**Table 1:** Comparison of sleep quality and quantity, LDL, HDL, TC and TG in exercise and control groups (pretest and posttest). Values are mean±SD changes in the parameter.

Parameter	Exercise	Control	P value
Difference in sleep quantity changes between control and exercise groups	6.23±2.67	9.50±1.47	0.005
Difference in sleep quality changes between control and exercise groups	228.28±192.93	100±74.02	0.037
Difference in changes of exercise group with control in LDL	8.56±14.76	7.57±4.85	0.007
Difference in changes of exercise group with control in HDL	0.78±2.61	0.42±0.78	0.728
Difference in changes of exercise group with control in TC	4.18±10.97	5.57±11.31	0.041
Difference in changes of exercise group with control in TG	23.81±25.82	11.57±12.8	0.001

There were no correlation between changing sleep quality and quantity with changes in TC, LDL, TG and HDL values after 10 weeks of exercise.

## DISCUSSION

Chronic debilitating diseases have several psychological sequelae. Renal diseases as well as hemodialysis and renal transplantation severely affect the physical and mental health of the patients. Our study showed that a period of exercise training can result in improved sleep quality and quantity in renal transplant recipients compared to a control group.

Exercise is believed to improve sleep. American Association of Sleep Disorders has recognized sports and physical activity as a major contributor to sleep hygiene, and cites exercise as a non-pharmacological intervention to improve sleep [36]. Studies on men and women of all ages indicate exercise as an effective approach to promote sleep status. In a meta-analysis of 12 studies, it was reported that regular exercises increase total sleep time and sleep slow waves [37]. However, in most studies, the effect of exercise on sleep indices has been evaluated in young people with an appropriate quality of sleep or in elite athletes. The results of our study showed that there was a significant correlation between the pre-treatment and post-treatment values of sleep quality and quantity—27% improvement in sleep quality and 30-minute increase in sleep duration. We found no other study similar to ours to evaluate the effect of exercise on sleep quality and quantity of renal transplant patients. However, with respect to studies on other subjects, our study was consistent with the results of investigations by King, *et al* (1997), Alaski, *et al* (2007), Abbey, *et al* (2008), and Joseph Norman, *et al* (2000), indicating significant changes in sleep quality and quantity due to aerobic activity and hiking. In our study, the duration of exercise was considered to be 10 weeks, while it was 21, 4 and 6 months in the studies by Abbey, *et al*, Alawski, *et al*, and Joseph Norman, *et al*, respectively. However, it can be observed that the sleep quality and quantity of the subjects has significantly been improved even after this relatively short period.

The mechanism of effect on sleep is so controversial that no simple analysis can be presented for it. Nevertheless, based on the available evidence, it can be stated that the melatonin hormone secreted by the pineal gland [38] has hypnotic effects by changing the central body temperature, and it is affected by exercise and physical activity [39]. It is noteworthy that secretion of melatonin is dependent upon exercise type and intensity, gender, age and exercise duration as intervening factors [40]. On the other hand, anabolic activity is stronger while asleep, and catabolic activity is higher during vigilance. Therefore, for proper balance of energy and maintenance of the balanced state of the body, high level of energy spent for physical activity should be counterbalanced in relaxation state. Therefore, the body will have a higher inclination to fall sleep [37]. And finally, physical activity may cause favorable changes in diurnal rhythm and increase the level of adenosine. All these improve sleep regulation and lead to maintenance of bodily energy [41].

Based on the results of this study, 10 weeks of exercise can significantly reduce the level of TC, LDL and TG but caused no significant change in HDL. It has been demonstrated that sports and physical exercise is an important weapon in fighting against high cholesterol levels and cardiac disease, and causes heart, bone and muscle reinforcement and eventually reduces body weight [42]. Rapidly growing data from human and animal studies confirm an important and effective role of exercise in prevention and treatment of cardiovascular diseases [43, 44]. Considering lack of research in this field, studies on other subjects showed that the results of our study were in agreement with those of Durstine, *et al*, who showed that exercise with 50% and 75% maximum oxygen consumption causes similar changes in decreasing cholesterol level. Kraus, *et al*, also observed the significant effect of physical activity with higher than 70% maximum heart rate on cholesterol level. So did Altena, *et al* [29], who showed the favorable effect of four weeks of exercise (five times per week) on plasma TC and LDL in seven non-qualified men and 11 women. Nash (2001), Fehlman (2002), Donovan, *et al* (2005), also showed that physical activity decreases LDL level. Patrick (2003), Tokmakidis, *et al* (2004), showed significant reduction in the level of TG by physical activity [45, 46] too. However, our findings were not consistent with the results of Stoedeflke, *et al*, Welsman *et al*, Brucha (2000), and Linda (2000) [47, 48]. With respect to no change in HDL level, our results were not in agreement with Kulie, *et al* [49], and Kumar, *et al* [50], findings but were in agreement with the results of Thomas, *et al* [51], Sungray (2002), and Kai (2003), who stated that resistive exercise causes no change in the level of HDL, and also in agreement with Welsman, *et al*’s study [52, 53]. Exercise type and duration, exercise conditions and age of subjects may be the reasons for the observed variances. The subjects of our study were renal transplant recipients, and physiological changes in patients seem to be different from healthy adults.

Several factors affect the changes in blood LDL levels, including the subjects’ sex, diet, medication, genetic characteristics, and duration of physical activity [54]. Several studies have acknowledged the beneficial effects of aerobic exercise in lowering LDL levels [55, 56]. Regarding the consumption of fat as a fuel both during activity and at the time of reset, physical exercise seems to be a factor decreasing the LDL levels. On the other hand, exercise increases the activity of sympathetic system and growth hormone level, which in turn activate lipolysis that can lead to loss of body fat [30]. Physical exercise increases the level of lipoprotein A and increases the level of LPL enzyme [56]. In addition, regular physical activity increases the transfer and use of TG by muscle. It has been shown that during and after exercise, plasma insulin level is decreased, which is probably one of the factors that can change the cholesterol level. Concurrent with the decrease in insulin level, glucagon secretion is increased, which accelerates the lipolysis rate from adipose tissue [47, 48]. With increasing LPL enzyme activity, it increases fatty acids release from adipose tissue and muscle, and increases the catabolism of TG and TG-rich lipoproteins, facilitating TG uptake from the blood [57], resulting in increased concentration of free fatty acids in plasma. LPL also catabolizes the lipid part of LDL. Therefore, the level of LDL in blood is expected to decrease [56]. Due to increased aerobic capacity and adaptations arising from the exercise in this study, it seems that the body prefers to provide its energy needs at rest and under maximum exercise from TG, which causes a reduced lipid profile [57]. It seems that the amount of exercise is an important stimulus, which can particularly affect the plasma concentration of lipids [30].

On the other hand, our findings showed that 10 weeks of exercise at 40% to 70% maximum heart rate caused a significant increase in HDL levels, which was consistent with a number of studies [58-60], but not with others [61, 62]. Gender, diet, medication as well as the intensity and duration of physical activity are among the factors that may affect the changes in HDL level [63, 64], although the mechanisms of HDL changes following physical activity are complex. Enzymes such as lipoprotein lipase, hepatic TG lipase and cholestryl ester carrier protein might play important roles in changing HDL concentrations [65]. In this study, the exercise intensity was lower than that required to increase HDL level, likely due to the weak physical status of the subjects who were not able of tolerating intensive exercise. However, increasing the number of sessions per week and the corresponding increase in the intensity of exercise may result in favorable changes and significant increase in the level of HDL too. 

The results of this study indicate no significant correlation between the changes in quantity and quality of sleep and lipid profile. Freeze, *et al* (2005), hypothesized that increasing muscle strength and improving sleep parameters are correlated with each other. Therefore, based on a study of the effect of six months of resistive exercises on sleep quality, they also assessed the muscle strength. However, no significant correlation was observed between improvement in sleep quality and increased muscle strength. Freeze, *et al*, stated the limited number of subjects as the reason for this observation [66], because there were only eight participants in their study. In our study, the number of participants in exercise and control groups was 29 and 15, respectively. Maybe the type of exercise training program has affected the results of this study in addition to the number of subjects. Canita, *et al* (2008), also observed that women who slept between six and seven hours were faced with a lower risk in terms of high TG and low HDL levels compared to women with less than five hours or equal to or more than eight hours of sleep [67].

In conclusion, renal transplant recipients are prone to dyslipidemia and sleep disorders because of taking multiple medications. Ten weeks of exercise training significantly improves the quality and quantity of sleep in renal transplant recipients and may also improve the lipid profile of these patients. Exercise can be a useful way to treat sleep disorders in renal transplant patients.
